# Practical approach to the diagnosis of adult-onset leukodystrophies: an updated guide in the genomic era

**DOI:** 10.1136/jnnp-2018-319481

**Published:** 2018-11-22

**Authors:** David S Lynch, Charles Wade, Anderson Rodrigues Brandão de Paiva, Nevin John, Justin A Kinsella, Áine Merwick, Rebekah M Ahmed, Jason D Warren, Catherine J Mummery, Jonathan M Schott, Nick C Fox, Henry Houlden, Matthew E Adams, Indran Davagnanam, Elaine Murphy, Jeremy Chataway

**Affiliations:** 1 Department of Molecular Neuroscience, UCL Institute of Neurology, London, UK; 2 Department of Neurology, Royal Free Hospital, London, UK; 3 Neurogenetics Unit, Neurology Department, Hospital das Clínicas da Faculdade de Medicina da Universidade de São Paulo, São Paulo, Brazil; 4 Department of Neuroinflammation, UCL Institute of Neurology, London, UK; 5 Department of Neurology, St Vincent's University Hospital University College Dublin, Dublin, Ireland; 6 Department of Neurology, Beaumont Hospital and Royal College of Surgeons in Ireland, Dublin, Ireland; 7 Memory and Cognition Clinic, Department of Clinical Neurosciences, Royal Prince Alfred Hospital and the Brain and Mind Centre, University of Sydney, Camperdown, New South Wales, Australia; 8 Dementia Research Centre, UCL Institute of Neurology, London, UK; 9 Lysholm Department of Neuroradiology, National Hospital for Neurology and Neurosurgery, London, UK; 10 Brain Repair & Rehabilitation, UCL Institute of Neurology, London, UK; 11 Charles Dent Metabolic Unit, National Hospital for Neurology and Neurosurgery Queen Square, London, UK

**Keywords:** neurogenetics, neuroradiology, dementia, adrenoleukodystrophy, movement disorders

## Abstract

Adult-onset leukodystrophies and genetic leukoencephalopathies comprise a diverse group of neurodegenerative disorders of white matter with a wide age of onset and phenotypic spectrum. Patients with white matter abnormalities detected on MRI often present a diagnostic challenge to both general and specialist neurologists. Patients typically present with a progressive syndrome including various combinations of cognitive impairment, movement disorders, ataxia and upper motor neuron signs. There are a number of important and treatable acquired causes for this imaging and clinical presentation. There are also a very large number of genetic causes which due to their relative rarity and sometimes variable and overlapping presentations can be difficult to diagnose. In this review, we provide a structured approach to the diagnosis of inherited disorders of white matter in adults. We describe clinical and radiological clues to aid diagnosis, and we present an overview of both common and rare genetic white matter disorders. We provide advice on testing for acquired causes, on excluding small vessel disease mimics, and detailed advice on metabolic and genetic testing available to the practising neurologist. Common genetic leukoencephalopathies discussed in detail include *CSF1R*, *AARS2*, cerebral arteriopathy with subcortical infarcts and leukoencephalopathy (CADASIL), and mitochondrial and metabolic disorders.

## Introduction

Adult patients with extensive white matter hyperintensities on MRI present the neurologist with a complex diagnostic task. There are a wide variety of disorders that can lead to these imaging appearances, including inflammatory, infective and malignant causes, as well as extensive small vessel disease. Often the most difficult patients to diagnose however are those with presumed genetic conditions, including the classical leukodystrophies, where the primary pathology is based in the myelin, or the genetic leukoencephalopathies, where the pathology is mainly neuronal or systemic. Patients may present with a wide spectrum of clinical features, including cognitive and neuropsychiatric changes, movement disorders, spasticity and seizures. The diversity of genetic aetiologies combined with the often-overlapping clinical and radiological phenotypes can make definitive diagnosis challenging.

In 2014 we set out a practical approach to navigate through the maze.^1^ However, even in the space of 5 years, there has been a substantial increase in the number of implicated genes. Improved phenotype–genotype correlation and increased access to advanced sequencing technologies have therefore changed our diagnostic approach.

This new review provides an opportunity to update and refine our practical approach for the diagnosis of genetic leukoencephalopathies in the era of whole genome sequencing. We would strongly recommend that it be read with the previous paper to avoid any repetition. As before, we emphasise the need to exclude as far as possible any acquired disorder, emphasise facets of the relatively commoner classical leukodystrophies/leukoencephalopathies, then highlight newer emerging diseases. Clinical/MRI features which point towards certain conditions are described. We end with an algorithm that we have found useful and describe our own experience.

For clarity, we are describing an approach where the symptoms have begun after the age of 16 and the MRI shows widespread cranial white matter change on standard (T2, fluid-attenuated inversion recovery (FLAIR)) sequences.

### Excluding common acquired leukoencephalopathies

**Figure 1:**
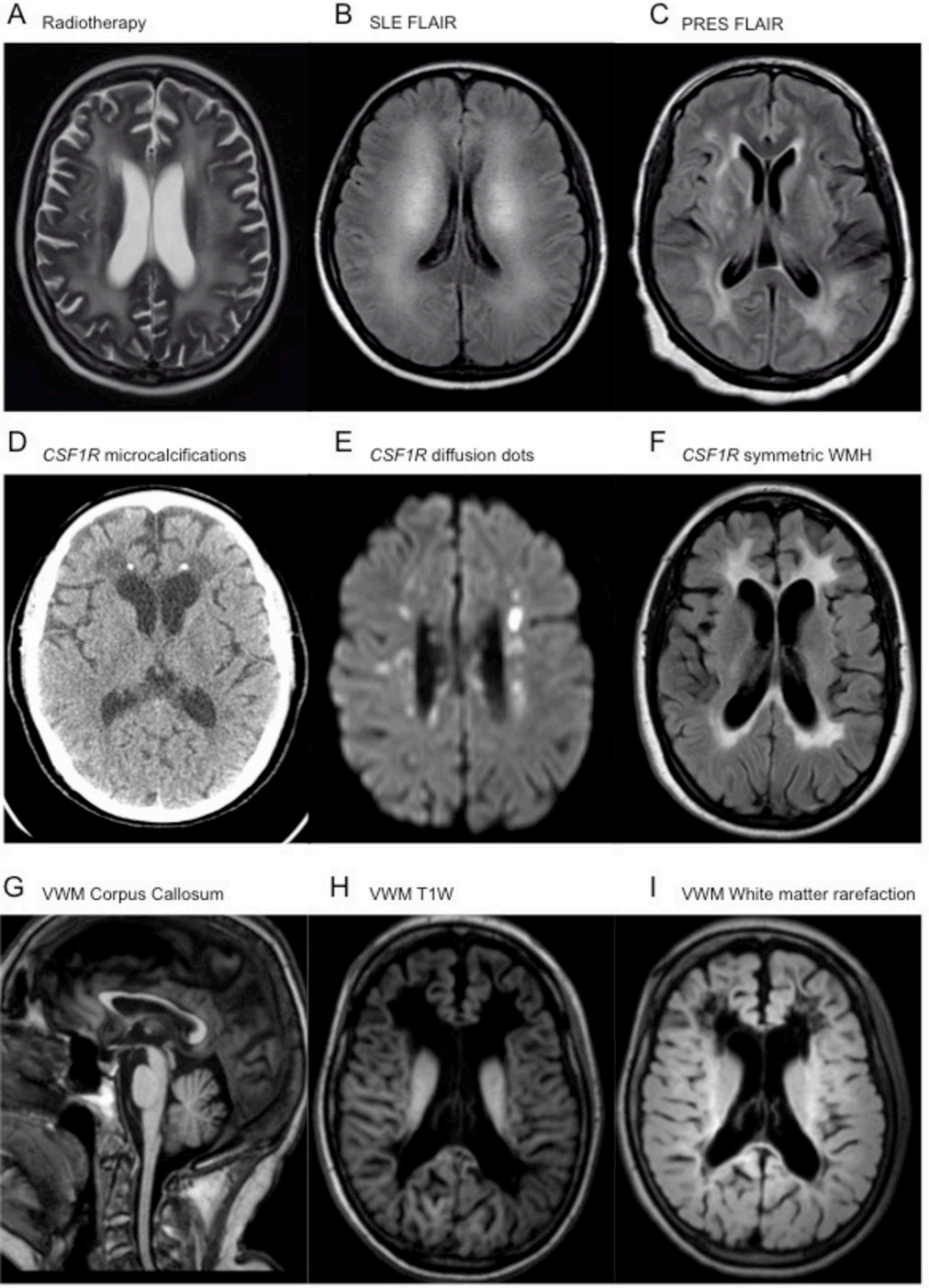
Axial T2-weighted MRI of radiotherapy changes (A), as well as axial FLAIR MRI of cerebral Systemic Lupus Eythematosus (SLE) (B) and PRES (C) demonstrating symmetrical and confluent periventricular and subcortical white matter signal hyperintensity (WMH). Axial non-contrast CT demonstrating subcortical volume loss, punctate calcification and confluent hypodensity adjacent to the frontal horns of the lateral ventricles (D). Axial B1000 DWI and FLAIR MRI acquisitions showing DWI avid punctate lesions (E) as well as confluent periventricular hyperintense signal changes (F) in *CSF1R* leukodystrophy. MRI sagittal T1W acquisition showing diffuse thinning of the corpus callosum (G), as well as axial T1W (H) and FLAIR (I) sequences demonstrating confluent rarefaction of the subcortical and periventricular white matter adjacent to the frontal horns and ventricular trigones of the lateral ventricles in VWM disease. FLAIR, fluid-attenuated inversion recovery; PRES, posterior reversible leukoencephalopathy syndrome; T1W, T1-weighted; VWM, vanishing white matter.

As emphasised previously it is imperative that treatable and acquired causes of white matter disease are ruled out as far as possible, before the patient is referred for investigation of a genetic leukodystrophy. As a minimum, all patients should undergo infective screening, including testing for HIV, syphilis, hepatitis B/C and tuberculosis. Patients with a history of immunosuppression should be tested for progressive multifocal leukoencephalopathy by cerebrospinal fluid (CSF) examination for the presence of JC virus. A high index of suspicion should remain for neoplasia, including primary central nervous system (CNS) lymphoma and gliomatosis cerebri. A careful history will reveal if the patient has been exposed to chemotherapy/radiotherapy (figure 1A) (in particular 5-fluorouracil and methotrexate) and recreational drugs like heroin or methanol, which can all lead to confluent, symmetrical leukoencephalopathy. Treatable inflammatory disorders like systemic lupus erythematosus must be considered (figure 1B,C). Posterior reversible leukoencephalopathy syndrome causes a dramatic leukoencephalopathy often with infarcts and microhaemorrhages (figure 1D), which improves over time. Further guidance on the initial work-up of these patients can be found in round 1 investigations (online supplementary table 1 in: Ahmed *et al*,^1^ *Journal of Neurology, Neurosurgery, and Psychiatry*).

Features suggestive of an acquired disorder include rapid onset, steroid responsiveness, systemic features, MRI gadolinium enhancement and cervical cord involvement—although of course there are exceptions as discussed below.

### Severe small vessel disease or genetic leukoencephalopathy?

A large number of patients referred for investigation will not be diagnosed with a genetic leukodystrophy, but instead will be diagnosed with severe acquired small vessel disease. These patients are more likely to present at an older age, have significant cardiovascular risk factors, and clinically are often asymptomatic or experience slow, indolent decline. They lack a clear family history to suggest a genetic origin. Imaging is likely to show increased T2/FLAIR signal in the periventricular and cerebral white matter, the basal ganglia, pons and cerebellum with chronic lacunes and cerebral microbleeds.^2^ In contradistinction to many inflammatory disorders such as multiple sclerosis, cervical cord imaging will be normal. In older patients (eg, >60 years), extensive genetic testing is unlikely to reveal a cause.^3^ However, in patients with this imaging appearance at a younger age, consideration should be given to the genetic forms of small vessel disease (eg, cerebral arteriopathy with subcortical infarcts and leukoencephalopathy (CADASIL), *COL4A1*), which are discussed below.

### Clinical and radiological pattern recognition

Many adult-onset leukoencephalopathies present in the same way, with a variable degree of cognitive decline, spasticity, apraxia and ataxia. This presentation is non-specific, and in these cases a non-biased approach using knowledge of the most frequent syndromes and next-generation sequencing technologies is the most efficient route to a diagnosis. However, many of the syndromes described below have particular clues to diagnosis which may be found in a careful history and neurological examination. The mode of inheritance is important and can limit the number of conditions to consider (table 1); however, the most common inheritance pattern in adults is sporadic. This may suggest an autosomal recessive mode of inheritance in a small family, reduced penetrance in an autosomal dominant condition or even a censored family history. The ethnicity of the patient and geographical region of origin are relevant as the frequency of each disorder can vary substantially in different regions.

**Table 1:** Leukoencephalopathies known to present in adulthood

Disorder	Inheritance	Investigation	Gene
Adult-onset leukodystrophies			
Inherited disorders of metabolism associated with abnormal biochemical tests			
X linked adrenoleukodystrophy	X linked	Very long chain fatty acids	*ABCD1*
Krabbe disease	AR	WCE (galactocerebrosidase)	*GALC, PSAP*
Metachromatic leukodystrophy	AR	WCE (arylsulfatase A) Urine sulfatides	*ARSA*
Cerebrotendinous xanthomatosis	AR	Serum cholestenol, urine bile alcohols	*CYP27A1*
Methylenetetrahydrofolate reductase deficiency	AR	Plasma amino acid profile	*MTHFR*
Homocystinuria	AR	Plasma amino acid profile	*CBS*
mtDNA mutations	Maternal	mtDNA sequencing Blood/cerebrospinal fluid lactate Muscle biopsy	Various

Disorder	Acronym	Inheritance	Gene
Genetic leukoencephalopathies			
Hereditary diffuse leukoencephalopathy with axonal spheroids and pigmented glia	HDLS	AD	*CSF1R*
Cerebral autosomal dominant arteriopathy with subcortical infarcts and leukoencephalopathy	CADASIL	AD	*NOTCH3*
Cerebral autosomal recessive cerebral arteriopathy with subcortical infarcts and leukoencephalopathy	CARASIL	AR	*HTRA1*
Cathepsin A-related arteriopathy with strokes and leukoencephalopathy	CARASAL	AD	*CTSA*
Cerebral leukodystrophy with retinal vasculopathy		AD	*TREX1*
Small vessel disease with ocular abnormalities		AD	*COL4A1*
Alexander disease		AD	*GFAP*
Adult-onset autosomal dominant leukodystrophy	ADLD	AD	*LMNB1 duplication*
*AARS2*-related leukodystrophy	*AARS2*-L	AR	*AARS2*
Vanishing white matter disease	VWM	AR	*EIF2B1-5*
Leukoencephalopathy with brainstem and spinal cord involvement and lactate elevation	LBSL	AR	*DARS2*
Mitochondrial neurogastrointestinal encephalopathy	MNGIE	AR	*TYMP*
Pelizaeus-Merzbacher disease	PMD	X linked dominant	*PLP1*
Pelizaeus-Merzbacher-like disease	PMLD	AR	*GJC2*
Adult polyglucosan body disease	APBD	AR	*GBE*
Hypomyelinating leukodystrophy with atrophy of the basal ganglia and cerebellum	H-ABC	AD	*TUBB4A*
Leukoencephalopathy with ataxia	LKPAT	AR	*CLCN2*
4H syndrome		AR	*POLR3A/POLR3B*
Labrune syndrome		AR	*SNORD118*
Gordon Holmes syndrome		AR	*RNF216*
Polycystic lipomembranous osteodysplasia with sclerosing leukoencephalopathy	PLOSL	AR	*TREM2* *TYROBP*

AD, Autosomal Dominant; AR, Autosomal recessive; WCE, White Cell Enzymes.

The most helpful and discriminating symptoms and signs are described in table 2. These include the importance of noting endocrine abnormalities such as premature ovarian failure (POF) (vanishing white matter (VWM) disease, *AARS2*) or hypogonadotrophic hypogonadism (Gordon Holmes syndrome, POLR3-related disorders). Movement disorders such as prominent parkinsonism or chorea are important, as are the presence of early autonomic (*LMNB1*) or urinary symptoms (adult polyglucosan body disease). Peripheral demyelinating neuropathy may suggest metachromatic leukodystrophy (MLD), and adrenal failure is highly suggestive of adrenoleukodystrophy (ALD).

**Table 2:** Clinical features that can be associated with specific leukodystrophies

Clinical characteristics of specific leukodystrophies	
Premature ovarian failure	VWM. Galactosaemia. AARS2-L.
Hypogonadotrophic hypogonadism	Gordon Holmes syndrome. 4H syndrome.
Parkinsonism	HDLS.
Chorea	Gordon Holmes syndrome.
Palatal myoclonus	Alexander disease.
Axonal peripheral neuropathy	ALD. LBSL. APBD. CTX.
Demyelinating peripheral neuropathy	MLD.
Optic atrophy	MLD. Krabbe disease. ALD. PMD mtDNA mutations.
Adrenal insufficiency	ALD.
Dental abnormalities	4H syndrome.
Tendon xanthomata	CTX.
Early urinary frequency	APBD.
Early autonomic features	ADLD.
Early prominent ataxia	CTX. Gordon Holmes syndrome. LKPAT.
Migraine with aura	CADASIL.
Cataracts	CTX mtDNA mutations.
Occasional rapid progression (<1 year)	ALD. Krabbe disease.

ADLD, adult-onset, autosomal dominant leukodystrophy; ALD, adrenoleukodystrophy; APBD, adult polyglucosan body disease; CADASIL, cerebral arteriopathy with subcortical infarcts and leukoencephalopathy; CTX, cerebrotendinous xanthomatosis; HDLS, hereditary diffuse leukoencephalopathy with spheroids; LBSL, leukoencephalopathy with brainstem and spinal cord involvement with elevated lactate; LKPAT, leukoencephalopathy with ataxia; MLD, metachromatic leukodystrophy; PMD, Pelizaeus-Merzbacher disease; VWM, vanishing white matter.

Similarly to the clinical presentations, the radiological appearance of genetic leukoencephalopathies can be non-specific, but there are important features to note. Deep white matter diffusion abnormalities have been well described in *CSF1R* and *AARS2*, in addition to prominent involvement of the corpus callosum. A patchy leukoencephalopathy with lacunes, microhaemorrhages and anterior temporal lobe involvement is suggestive of a vascular disorder such as CADASIL or cathepsin A-related arteriopathy with strokes and leukoencephalopathy (CARASAL). Hypomyelination is an important sign to note, as there are only a small number of genetic diseases known to cause this radiological appearance. Useful radiological signs can be found in table 3.

**Table 3:** Imaging features associated with specific leukodystrophies

Imaging characteristics of specific leukodystrophies	
Frontal predominance	Alexander disease.
Basal ganglia abnormalities	H-ABC mtDNA mutations. PLOSL (calcifications).
Hypomyelination	PMD/PMLD. H-ABC. 4H syndrome.
Anterior temporal lobe signal abnormality	CADASIL. CARASIL. CARASAL.
Deep white matter diffusion dots	HDLS. AARS2-L.
Corpus callosum involvement	HDLS. AARS2-L. HSP genes (*SPG11, SPG15, Fa2H*).
’Tadpole brainstem’ (atrophy of the cervical cord and medulla with preserved pons)	Alexander disease.
Dentate nucleus signal change/cysts	CTX.
Spinal cord abnormalities	Adrenomyeloneuropathy. LBSL. APBD. Alexander disease.
Middle cerebellar and cerebral peduncles	LKPAT.
Contrast enhancement	ALD. Alexander disease. Krabbe disease.
Periventricular microcalcifications (visible on CT)	HDLS.
Extensive calcifications and cysts	Labrune syndrome.
Bone cysts on X-ray	PLOSL.

ALD, adrenoleukodystrophy; APBD, adult polyglucosan body disease; CADASIL, cerebral arteriopathy with subcortical infarcts and leukoencephalopathy; CARASAL, cathepsin A-related arteriopathy with strokes and leukoencephalopathy; CARASIL, cerebral autosomal recessive arteriopathy with subcortical infarcts and leukoencephalopathy; CTX, cerebrotendinous xanthomatosis; H-ABC, Hypomyelination with atrophy of the basal ganglia and cerebellum; HDLS, hereditary diffuse leukoencephalopathy with spheroids; HSP, Hereditary Spastic Paraplegia; LBSL, leukoencephalopathy with brainstem and spinal cord involvement with elevated lactate; LKPAT, leukoencephalopathy with ataxia; PLOSL, polycystic lipomembranous osteodysplasia with sclerosing leukoencephalopathy; PMD, Pelizaeus-Merzbacher disease; PMLD, Pelizaeus-Merzbacher-like disorder.

### Metabolic leukodystrophies

After excluding common acquired leukoencephalopathies, the first priority should be to identify patients with an inherited metabolic disorder. A small number of tests will detect the diseases that present in adulthood. Recommended testing includes a very long chain fatty acid (VLCFA) profile in men (ALD), specific white cell enzyme activities (Krabbe disease, MLD), raised serum cholestanol/urinary bile alcohols (cerebrotendinous xanthomatosis (CTX)) and plasma amino acids (methylenetetrahydrofolate reductase (MTHFR) deficiency and homocystinuria) (table 1).

### X linked ALD

Adult-onset ALD is a rare X linked metabolic disorder of peroxisomal fatty acid beta-oxidation which results in the accumulation of VLCFA in plasma and tissues, including white matter and the adrenal cortex.^4^

ALD is associated with three main phenotypes, including Addison-only disease, adrenomyeloneuropathy (AMN) and cerebral ALD (CALD). Addison-only presents most commonly by age 8 years, and although it begins without evidence of neurological abnormality some degree of disability (most commonly AMN) usually develops later. Indeed, virtually all patients with ALD who reach adulthood develop AMN, usually between 20 and 30 years of age. AMN is a non-inflammatory distal axonopathy, and is characterised by progressive spastic paraparesis, sensory ataxia, sphincter dysfunction, impotence and pain. CALD usually only affects men and presents with rapidly progressive inflammatory demyelination in the brain, leading to rapid cognitive and neurological decline, dementia, ataxia, seizures and death.

MRI is always abnormal in neurologically symptomatic men with cerebral disease. Early changes include T2/FLAIR hyperintensities in the parieto-occipital regions and splenium of the corpus callosum. A minority of patients will demonstrate signal abnormalities predominantly in the frontal lobes and genu of the corpus callosum. Peripheral rim enhancement is a typical feature.^4 5^

Elevated levels of VLCFAs in the blood are suggestive of a peroxisomal disorder, and the diagnosis of ALD can be confirmed by sequencing of the *ABCD1* gene. Early diagnosis and family screening are essential to reduce the risk of untreated hypoadrenalism, and to identify early those patients who may benefit from haematopoietic stem cell transplantation (HSCT).^6^

### Krabbe disease

Krabbe disease is a rare autosomal recessive disorder caused by loss of function mutations in *GALC,* leading to deficiency of galactocerebrosidase, a lysosomal enzyme responsible for the degradation of galactocerebroside to ceramide and galactose. In Krabbe disease, galactocerebroside accumulates in the peripheral and central nervous system producing cerebral atrophy, loss of myelin, gliosis and globoid cells.^7^

There are three forms of Krabbe disease: infantile, juvenile and adult. The infantile form is the most severe and usually presents between 3 and 6 months of age. After a normal neonatal period, those affected develop a rapidly progressive course involving irritability, hyperaesthesia, visual and hearing loss, severe cognitive and motor deterioration, and seizures. This group rarely survives beyond 2 years. Nerve conduction studies show a demyelinating peripheral neuropathy and CSF analysis may reveal increased total protein concentration.^8^

Juvenile and adult (late) forms of the disease have a milder, more varied phenotype, a slower rate of progression and a significantly longer lifespan (although we have observed a rapid deterioration over a year in one case). Typical features include spasticity, dementia, ataxia, peripheral neuropathy and visual loss. The T2/FLAIR signal abnormality in Krabbe disease predominantly affects the corticospinal tracts, from the cortex, through the corona radiata internal capsules and cerebral peduncles.^9^ The optic radiations are frequently involved,^9^ and intracranial calcifications have been reported, but may only be apparent on CT imaging.^10^

The diagnosis of Krabbe disease can be made by assay of galactosylceramidase activity via white cell enzyme testing.

### Metachromatic leukodystrophy

MLD is an autosomal recessive disorder caused mainly by deficient activity of arylsulfatase A (ARSA). ARSA is responsible for the desulfation of cerebroside sulfate, a major glycolipid of myelin, and decreased ARSA activity leads to the accumulation of cerebroside sulfate in the CNS and peripheral nerves (as well as the kidneys and other visceral organs). The result is central and peripheral demyelination.^11^

In almost all cases, recessive mutations in the *ARSA* gene are responsible, although very rarely, MLD can be caused by mutations in the *PSAP* gene.^12^ Three major subtypes exist—late infantile (age 6 months to 2 years), juvenile (age 3–16 years) and adult (age >16). Peripheral neuropathy occurs in all forms and gallbladder involvement (hyperplastic polyps) is also common. Late infantile onset is associated with poor prognosis (death typically occurs within 5–6 years) and manifests as regression of motor skills, gait abnormalities, seizures, ataxia, hypotonia, extensor plantars and optic atrophy. Juvenile disease presents similarly but is more heterogeneous. Progression is slower, and these children may survive until early adulthood. Adult onset is usually heralded by dementia, behavioural difficulties, and in a minority with psychosis.^13^

Nerve conduction studies show marked slowing. Brain MRI reveals symmetric periventricular white matter lesions and cortical atrophy, often with a tigroid or stripe pattern, caused by the appearance of the spared perivascular white matter.^14^ Diagnosis is established by demonstrating deficient ARSA activity in leucocytes (white cell enzyme testing) or cultured skin fibroblasts. Clinicians should be aware of the pseudodeficiency state, in which ARSA activity levels are low, but do not cause disease. In pseudodeficiency, the activity level is typically 5%–20% of controls.^15^ In affected individuals with MLD, urine sulfatide excretion is increased, generally tenfold to a hundred-fold greater than controls. Urine sulfatide excretion may therefore be used to differentiate pseudodeficiency from deficiency. However, the assay for urine sulfatide excretion is not currently routinely available in the UK and, as testing for the pseudodeficiency variants can be done rapidly, this is the usual first-line test.

No curative treatment is currently available, but HSCT has slowed disease progression in some patients.^16^ Other promising novel treatments include gene therapy and enzyme replacement.^17^

### Cerebrotendinous xanthomatosis

CTX is an autosomal recessive disorder of bile acid synthesis caused by mutation of the cytochrome P450 gene *CYP27A1*, resulting in the production of defective sterol 27-hydroxylase. Consequently, CTX is associated with high levels of cholestanol in plasma and its accumulation in tissue. This gives rise to hallmark clinical manifestations of chronic diarrhoea, bilateral cataracts, tendon xanthomas or tendon thickening, and neurological dysfunction.^18^

Typical neurological manifestations can include intellectual disability, autism, behavioural and psychiatric problems, dementia, ataxia and epilepsy. MRI studies show cerebral and cerebellar atrophy, extensive white mater lesions of the spinal cord, and bilateral T2/FLAIR hyperintensity of the dentate nuclei and surrounding white matter.

Cholestanol concentrations are increased in plasma, brain, xanthomas and bile. Increased quantities of bile alcohols are useful as a secondary diagnostic test. CSF levels of cholestanol, cholesterol, apolipoprotein B, apolipoprotein A1 and albumin are increased.^18^

Treatment is with chenodeoxycholic acid (a cholesterol 7α-hydroxylase enzyme inhibitor), which is effective in improving biochemical findings, and in those without advanced disease may improve or stabilise neurological features.^19^

### MTHFR deficiency

Autosomal recessive mutations in the *MTHFR* gene cause a spectrum of symptoms, with onset ranging from childhood to adulthood. Biochemically, there are elevated plasma homocysteine, homocystinuria and low methionine levels. Patients may develop seizures, cognitive decline, recurrent thrombotic stroke and leukoencephalopathy. The neurological signs may improve with treatment, the mainstay of which is folic acid and betaine supplementation.^20^

### Genetic leukoencephalopathies

Since our previous review, a number of conditions have gained in prominence or have been newly described. They are diagnosed genetically, although as shown in tables 2 and 3 there can be typical diagnostic features to focus the genetic analysis.

### CSF1R

Mutations in the *CSF1R* (colony stimulating factor-1 receptor) gene are known to cause an adult-onset leukodystrophy termed hereditary diffuse leukoencephalopathy with spheroids (HDLS).^21^ This condition has been shown to be one of the most common causes of adult-onset leukodystrophy, accounting for approximately 10% of cases.^22^ First described in a large Swedish kindred,^23^ this is an autosomal dominant disorder in which affected members develop a clinical course characterised by dementia, psychiatric changes and motor decline. The age of onset is variable, even within families, but typically patients develop symptoms in their 40s (range 18–78 years). The penetrance of the disorder is not known, but is likely to be high. Because of incomplete penetrance (and the possibility of de novo mutations), some affected patients will not have a family history and may appear to have a sporadic disorder.

The condition is found worldwide and has a well-described clinical phenotype. Prominent symptoms include parkinsonism,^24^ which is usually not levodopa-responsive, and upper motor neuron signs such as spasticity and ataxia. A recent large review found the mean disease duration to be 6.8 years from symptom onset to death.^25^

Pathologically the disease is characterised by the presence of astrogliosis with myelin and axonal loss and frequent axonal spheroids in the cerebral white matter. The spheroids are visible with H&E staining, but also stain positive for neurofilaments, p62, amyloid precursor protein APP and β amyloid. There are typically pigmented microglia present which are autofluorescent and stain positive for CD68. The pathological appearance of this disorder is relatively specific and lends the disease its name. Prior to the identification of the responsible gene, the diagnosis of HDLS could only be confirmed pathologically.

The MRI appearance of HDLS is consistent, with typical features including confluent, largely symmetric T2 hyperintense/T1 hypointense signal abnormality in the frontoparietal and periventricular white matter, which spares the U-fibres, and involves the pyramidal tracts and corpus callosum.^26^ Punctate areas of restricted diffusion may be present (termed deep white matter diffusion dots) and their appearance may even mimic CNS vasculitis.^22^ Calcifications may be present, particularly in the periventricular white matter adjacent to the frontal horns (figure 1D–F).

It was shown in 2011 that HDLS is largely caused by mutations in the *CSF1R* gene.^21 27^ Almost all mutations are found in the tyrosine kinase domain of the *CSF1R* protein and the majority of mutations are missense. A small number of splice site, frameshift and indel mutations have also been identified.

### AARS2

Mutations in *AARS2* (alanyl-transfer (t)RNA synthetase 2) are emerging as a rare cause of leukodystrophy with similar clinical, imaging and radiological phenotype to *CSF1R* mutations.^28 29^ This is an autosomal recessive disorder, but frequently appears sporadic, with a younger age of onset than *CSF1R* (mean age 29 years, range 15–44 years). However the initial symptoms are very similar, with psychiatric changes and cognitive decline, parkinsonism, pyramidal signs, ataxia and seizures. Almost all female cases have experienced POF, although no endocrine or reproductive abnormalities are found in men. MRI features include largely symmetric and confluent T2 hyperintense/T1 hypointense white matter signal change in the frontoparietal white matter, corpus callosum and pyramidal tracts. There are punctate areas of restricted diffusion on diffusion weighted imaging (DWI) often running parallel to the ventricles. A distinguishing feature from *CSF1R* is that the white matter signal suppresses on FLAIR imaging, indicating a degree of rarefaction.^26^

The clinical picture over time remains similar to *CSF1R*, with early cognitive symptoms followed by severe and rapid motor decline. Most patients are fully dependent within 5 years of symptom onset. In one case, pathological features of HDLS were found on brain biopsy, with axon and myelin loss and frequent axonal spheroids containing neurofilament, p62, APP and β amyloid. Pigmented microglia were also seen, and the pathological features were felt to be indistinguishable from HDLS. In another case, no pathological abnormalities were found in a brain biopsy,^29 30^ and further pathological descriptions are required.

*AARS2* encodes a t-RNA synthetase responsible for correctly loading alanine onto tRNA-ala for the translation of mitochondrial proteins. Most commonly patients will have compound heterozygous *AARS2* mutations, although homozygous cases have also been reported. Loss of function mutations (frameshift, nonsense, splice site) are the most common, but there are some pathogenic missense mutations, including the recurrent R199C mutation. Mutations in this class of genes (tRNA synthetases) are increasingly implicated in a diverse range of neurological disorders.^31 32^

### VWM disease

VWM disease is an autosomal recessive disorder characterised by progressive neurological impairment and the cystic degeneration of white matter that is visible on MRI. It most commonly presents in childhood, but many adult-onset cases have been reported. The most frequent presenting features include ataxia, spasticity, seizures and cognitive decline. Often patients will experience episodes of severe neurological deterioration after a minor insult, such as a head trauma, an infection or even emotional distress.^33^ An additional feature in women with VWM is POF; hence, VWM is sometimes referred to as ovarioleukodystrophy.

In one study of adult-onset VWM, the mean age of onset was found to be 31 years, with clinical features including neurological and psychiatric presentations. Frequent MRI features included cerebral and cerebellar atrophy and cavitating leukoencephalopathy with involvement of the corpus callosum.^34^ The characteristic feature is that the periventricular white matter takes on the same signal intensity as the CSF on T2-weighted and FLAIR imaging (figure 1G–I). VWM is caused by homozygous and compound heterozygous mutations in any of the five genes that encode the subunits of the translation initiation factor EIF2B (*EIF2B1–EIF2B5*).^35^

### Vascular leukoencephalopathies

A number of different genes come under the umbrella term of vascular leukoencephalopathies, including *NOTCH3* (CADASIL), *HTRA1* (cerebral autosomal recessive arteriopathy with subcortical infarcts and leukoencephalopathy (CARASIL)), *CTSA* (CARASAL), *COL4A1* and *TREX1*.

### Cerebral arteriopathy with subcortical infarcts and leukoencephalopathy

**Figure 2:**
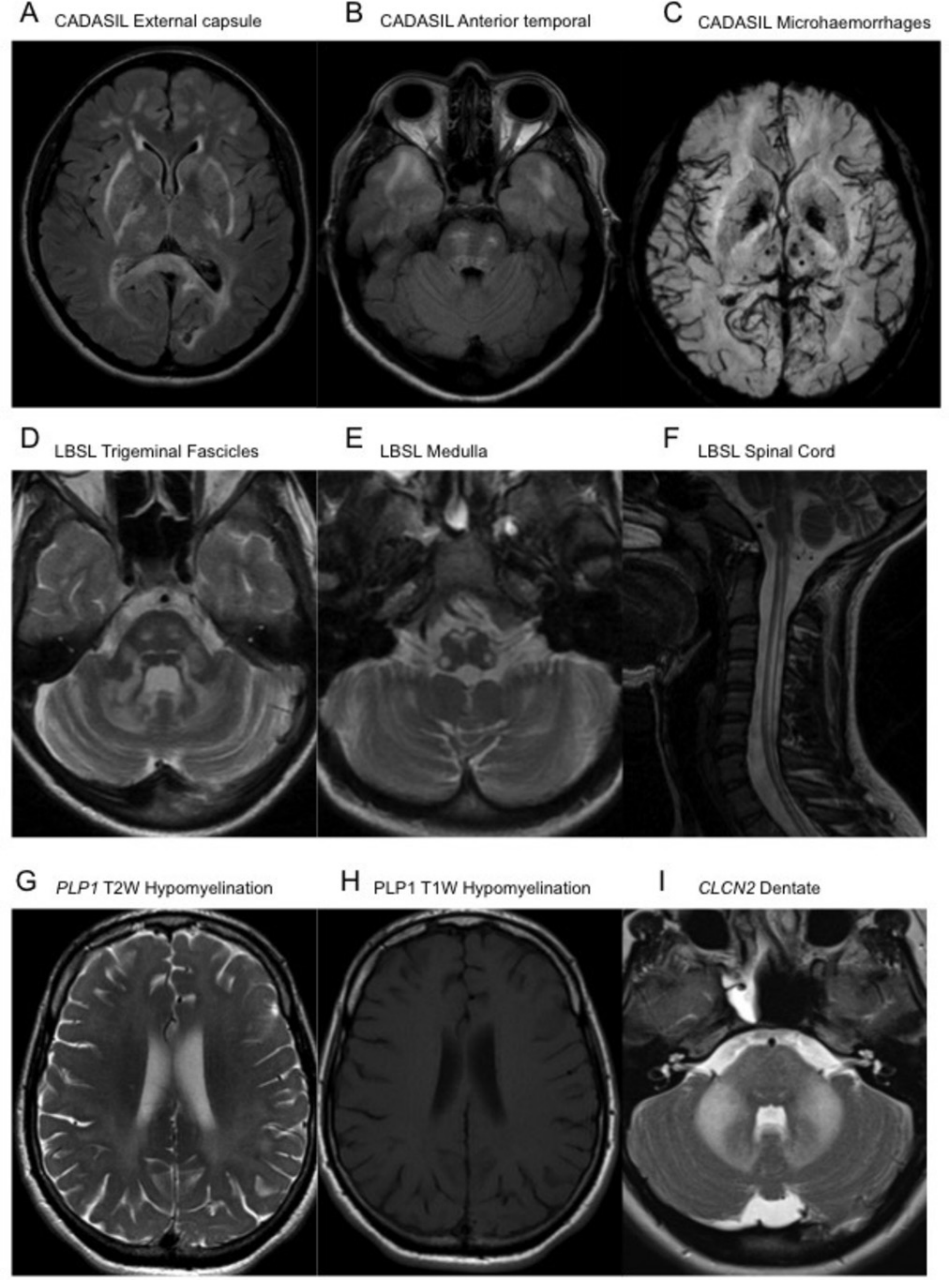
Axial FLAIR MRI sequences demonstrating confluent signal hyperintensity involving the external capsules, posterior limb of the internal capsules, peritrigonal white matter and splenium of the corpus callosum (A), as well as of the subcortical white matter of the temporal poles and central pons (B) in CADASIL. Minimal intensity projection from a susceptibility weighted imaging acquisition MRI demonstrating multiple punctate foci of paramagnetic susceptibility limited within the thalamus, left putamen and subcortical white matter of the left occipital lobe (C), in keeping with multiple microhaemorrhages in CADASIL. Axial T2W MRI sequences demonstrating signal hyperintensity within the trigeminal fascicles and cerebellar white matter (D), as well as within the pyramids, decussation of the medial lemnisci and inferior cerebellar peduncles at the level of the medulla oblongata (E) in LBSL. A sagittal T2W sequence of the upper spinal cord in the same patient demonstrates contiguous longitudinally extensive signal hyperintensity of the dorsal columns and lateral cortical spinal tracts (F). Axial T2W (G) and T1W (H) MRI sequences in a patient with Pelizaeus-Merzbacher disease illustrating the confluent diffuse T2W hyperintense signal within the white matter of the cerebrum, appearing unremarkable on the T1W sequences, suggestive of hypomyelination. Axial T2W sequences demonstrating confluent hyperintense signal change within the pons, middle cerebellar peduncles and dentate nuclei in a patient with a *CNCL2* leukodystrophy (I). CADASIL, cerebral arteriopathy with subcortical infarcts and leukoencephalopathy; FLAIR, fluid-attenuated inversion recovery; T1W, T1-weighted; LBSL, leukoencephalopathy with brainstem and spinal cord involvement with elevated lactate; T2W, T2-weighted.

CADASIL is caused by heterozygous mutations in the *NOTCH3* gene. The condition is autosomal dominant and is characterised by recurrent stroke at a young age, cognitive decline, migraine with aura and depression.^36^ The mean age of first stroke is 46 years and typically the events are subcortical, ischaemic lacunes. Reversible episodes of encephalopathy may also occur. On brain MRI, the first abnormality detected is often white matter signal abnormality in the temporal poles. Over time the burden of white matter lesions increases, with the periventricular, frontoparietal and external capsular white matter most affected.^37^ There are frequently dilated perivascular spaces and subcortical lacunes. Microhaemorrhages may be detected on gradient echo imaging (figure 2A–C). The diagnosis is supported by the finding of granular osmiophilic material in small arterioles by electron microscopic examination of tissue, usually a skin biopsy. Pathogenic *NOTCH3* mutations lead to the gain or loss of a cysteine residue in one of the epidermal growth factor-like repeat (EGFr) domains of the *NOTCH3* protein. These EGFr domains normally contain six cysteine residues, and any alteration in this number has been shown to lead to *NOTCH3* aggregation. *NOTCH3* mutations are not fully penetrant, but it has been shown that cysteine altering mutations affecting the first six EGFr domains are the most penetrant and are associated with the highest burden of white matter disease.^38^

### Cerebral autosomal recessive arteriopathy with subcortical infarcts and leukoencephalopathy

This autosomal recessive disorder is caused by biallelic mutations in the HTRA1 gene. CARASIL was first identified and is most common in Japan, but cases in Europe and South America have also been identified.^39 40^ Clinically it is characterised by dementia, parkinsonism, upper motor neuron signs, and extraneurological features including alopecia and back pain with spondylosis deformans. Acute strokes are common and the typical age of onset is the teenage years to 20s. MRI appearance is similar to CADASIL (above), with spondylosis deformans potentially visible on spinal imaging.^41^

Recently, it has been proposed that heterozygous HTRA1 mutations can also lead to autosomal dominant severe small vessel disease. In one study these patients were termed ‘manifesting heterozygotes with CARASIL’ and were described with a similar but less severe phenotype to recessive mutations.^42^ Spondylosis was found in all manifesting heterozygotes. However, another recent study found that the phenotype of heterozygous *HTRA1* mutations was different from both CADASIL and CARASIL, and no extraneurological features were observed, although the imaging appearance was similar.^43^

### Cathepsin A-related arteriopathy with strokes and leukoencephalopathy

This very recently described entity due to heterozygous mutations in the *CTSA* gene is named cathepsin A-related arteriopathy with strokes and leukoencephalopathy (CARASAL). The initial report described two Dutch families, distantly related, in whom members were affected by a severe leukoencephalopathy, accompanied often by ischaemic or haemorrhagic stroke, therapy-resistant hypertension and later cognitive decline.^44^ The MRI pattern was very similar to CADASIL. Since the initial report, another case has been described from the UK with identical imaging and a similar phenotype, although with additional prominent brainstem features reported.^3 45^ To date, all patients described have carried the same R325C mutation in *CTSA*. It is unclear whether this is the only mutation in *CTSA* which is causal for CARASAL or whether further mutations will be identified.

### *COL4A1* and *TREX1*

Both *COL4A1* and *TREX1* are associated with leukoencephalopathies in which ocular or retinal abnormalities may be present. *COL4A1* is characterised by diffuse leukoencephalopathy, often with dilated perivascular spaces and microhaemorrhages, and retinal abnormalities including arteriolar tortuosity or retinal haemorrhage.^46^ It is an autosomal dominant disorder and shows incomplete penetrance. Additional CNS features include intracerebral haemorrhage, calcifications and cerebellar atrophy. Ocular abnormalities are not exclusively confined to the retina in *COL4A1* with anterior segment dysgenesis, congenital cataract and nystagmus all reported.^47^ A second form of leukoencephalopathy with retinal vasculopathy is caused by heterozygous frameshift mutations in the c-terminus of the *TREX1* gene.^48^ In addition to the microvascular disease of the retina which can lead to visual loss, Raynaud’s phenomenon and migraine are both frequently found.^49^ A phenotype of *TREX1* mutations called hereditary endotheliopathy with retinopathy, nephropathy and stroke may present with progressive, contrast-enhancing lesions with surrounding oedema, which may be mistaken for tumours.^50^ Calcifications are found in more than 50% of patients.^51^

### Mitochondrial DNA mutations

Mitochondrial DNA mutations are a rare cause of adult-onset leukoencephalopathy. Typically patients will present with a multisystem disorder, often including short stature, migraine, sensorineural deafness, cardiac defects and exercise intolerance. There is often involvement of the deep grey structures on imaging, such as the basal ganglia and thalamus, but symmetric and confluent white matter abnormalities can occur.^52^ Stroke-like episodes or cortical blindness would point to mitochondrial encephalopathy with lactic acidosis and stroke-like episodes (m.3243A>G); myoclonus, seizures and ataxia are suggestive of mitochondrial encephalopathy with ragged red fibres (m.8344A>G). Periventricular signal abnormalities have been reported in Leber’s hereditary optic neuropathy, but only as a minor feature.^53^ Common mitochondrial point mutations can be readily screened in blood, but if these are negative and a suspicion of mitochondrial disease remains, next-generation sequencing of the complete mitochondrial genome from the muscle may be necessary. Clinicians should be aware that exome sequencing and panel-based sequencing typically target only *nuclear* genes, that is, *not* mitochondrial DNA. There are methods to extract mitochondrial DNA sequence from exome data, but this is not commonly done.

### Nuclear encoded mitochondrial genes

There are a large number of nuclear encoded genes with established or putative mitochondrial functions (>1000 in MitoCarta).^54^ This section could never be exhaustive, and some genes, such as *AARS2* and *DARS2*, are discussed elsewhere in detail. Many of these disorders are only known to affect children, but two disorders of mtDNA maintenance can present with prominent leukoencephalopathy in adulthood, namely mitochondrial neurogastrointestinal encephalomyopathy (MNGIE) and *POLG* mutations. MNGIE is an autosomal recessive disorder caused by mutations in the *TYMP* gene.^55^ It usually presents in adulthood with ptosis, chronic progressive external ophthalmoplegia, gastrointestinal dysmotility including pseudo-obstruction, peripheral neuropathy, and diffuse leukoencephalopathy. A similar disorder can be caused by mutations in *POLG*, although these cases usually have less prominent leukoencephalopathy.^56^ Involvement of the pulvinar of the thalamus has been reported in *POLG* mutations.^57^ Nuclear encoded mitochondrial gene defects can be identified by whole exome or panel sequencing. Variants, either single nucleotide variants or deletions, of mtDNA are usually identified by specific mtDNA sequencing from affected tissue, often muscle.

### Leukoencephalopathy with brainstem and spinal cord involvement with elevated lactate

Leukoencephalopathy with brainstem and spinal cord involvement with elevated lactate (LBSL) is an unusual and rare leukoencephalopathy caused by autosomal recessive mutations in the *DARS2* gene.^58^ Usually patients develop symptoms in childhood, but the condition tends to be slowly progressive and evolves over time, so the diagnosis is usually made when the patient is an adult. The most common presentation consists of childhood onset of distal weakness and wasting, with pes cavus, and initially patients may be given a diagnosis of hereditary sensory motor neuropathy. Over time, upper motor neuron signs including spasticity and extensor plantar responses develop. Slowly progressive ataxia is another common feature, and is due to both cerebellar involvement and dorsal column loss. The neuroimaging appearance of LBSL is quite distinct, with a diffuse T2 hyperintense signal abnormality of the cerebral and cerebellar white matter, corpus callosum, medullary pyramids, medial lemniscus, intraparenchymal course of the trigeminal nerves, superior, middle and inferior cerebellar peduncles, and the dorsal columns and lateral corticospinal tracts of the spinal cord. A lactate peak is seen on magnetic resonance spectroscopy, but blood and CSF lactate are not elevated^59^ (figure 2D–F). *DARS2* mutations are usually compound heterozygous, and the most frequent variant is a splice site mutation in intron 2.^58^ This region is poorly covered by next-generation sequencing approaches, and single-gene testing is recommended if the clinical and radiological picture is suggestive of LBSL.

### Alexander disease

Caused by heterozygous mutations in *GFAP*, Alexander disease has infantile, juvenile and adult onset forms.^60^ Mutations are often de novo, and young children typically present with seizures, motor regression and macrocephaly. *GFAP* encodes the glial fibrillar acidic protein, an essential component of astrocytes. Alexander disease is characterised pathologically by the presence of Rosenthal fibres, which are long, filamentous eosinophilic fibres largely composed of GFAP protein.^61^

In infantile cases, death occurs before 2 years. In juvenile cases, onset is between 4 and 10 years and is typified by ataxia, cognitive decline and bulbar symptoms. Survival can be up to 20–30 years. Adults tend to present with bulbar or pseudobulbar palsy, spasticity, ataxia, cognitive decline and dysautonomia.

The MRI pattern in Alexander disease often falls into two extremes. In most infantile and juvenile cases, there is extensive white matter abnormality with a frontal predominance. In adults, however, the abnormalities are often restricted to the posterior fossa, particularly atrophy of the medulla and cervical spinal cord. Often the lesions are contrast-enhancing, or there may be contrast enhancement of the ventricular lining or periventricular rim.^62^

### *LMNB1* duplication

Duplication of *LMNB1* causes a form of adult-onset, autosomal dominant leukodystrophy (ADLD) characterised by autonomic dysfunction, spasticity, ataxia and cognitive decline. Symptoms usually develop in the fifth or sixth decade.^63^ Autonomic symptoms may be prominent and include orthostatic hypotension, urinary incontinence, constipation and erectile dysfunction. MRI features include T2W/FLAIR hyperintensity of the subcortical and deep cerebral white matter, the cerebellar peduncles, the pyramidal tracts and brainstem.^64^ It is important to note that copy number variants are not reliably identified by next-generation sequencing. Therefore, if ADLD is suspected clinically, specific testing for *LMNB1* duplications should be performed.

### Hypomyelinating disorders (*PLP1*, *GJC2*, *TUBB4A*, *POLR3A*, *POLR3B*, *CLCN2*, *NKX6-2*)

Hypomyelination is an extremely useful radiological sign, and in adults can significantly reduce the number of potential genes to test. Hypomyelination should be considered where the T2/FLAIR hyperintensity is diffuse, and often seems to be affecting all of the white matter uniformly. In contrast to demyelinating disorders where the white matter signal is hypointense on T1 imaging, in hypomyelination it may be isointense, or mildly hypointense or hyperintense.^65^ This sign is easily overlooked if the T1 sequences are not examined (figure 2G–H).

The most common cause of hypomyelinating leukoencephalopathy is Pelizaeus-Merzbacher disease (PMD), caused by mutations in the proteolipid gene *PLP1*. The most common presentations of PMD include the connatal form, where symptoms are present at the time of birth, and the classical form, where symptoms develop within the first months of life.^66^ Symptoms begin with nystagmus, hypotonia, ataxia and head tremor. Later motor and language development is delayed. *PLP1* duplications are the most common mutation type in children. As *PLP1* is an X linked gene, typically male patients are more severely affected than female patients. The connatal and classical forms are not seen in female patients. However, female *PLP1* mutation carriers can develop symptoms in adulthood. The phenotype in women often consists of slowly progressive spastic paraplegia and this presentation has been assigned SPG2.^67^ The most common mutation type in female carriers who manifest symptoms is a nonsense mutation.^68^ Adult-onset disease in male patients is more aggressive, with prominent head tremor, ataxia, spasticity and cognitive decline. MRI in both male and female patients shows a diffuse hypomyelinating leukodystrophy which is hyperintense on T2/FLAIR and often isointense or mildly hyperintense on T1. The pattern can resemble tiger stripes (tigroid pattern).

Autosomal recessive mutations in *GJC2* are a cause of a Pelizaeus-Merzbacher-like disorder.^69^ The phenotype is similar to that of *PLP1* mutations but male and female patients are equally affected. Like *PLP1*, most patients have onset of symptoms in early life, but there are reports of later onset with slow progression into adulthood, again mostly manifesting by spastic paraplegia, cerebellar signs and a hypomyelinating picture on MRI.^70^

Autosomal recessive mutations in *CLCN2* are a very rare cause of adult-onset leukoencephalopathy, with findings mainly limited to the posterior limb of the internal capsules, the dentate nucleus, the cerebral peduncles and middle cerebellar peduncles (figure 2I). There may be evidence of restricted diffusion in these areas. There may be more extensive involvement of cerebellar white matter, and cerebral white matter may demonstrate features of hypomyelination. In adults, the most common presentation is mild cerebellar ataxia.^71^

A number of other genes have been associated with hypomyelination, including *TUBB4A* (with basal ganglia abnormalities),^72^ *POLR3*-related disorders (dental abnormalities and hypogonadotrophic hypogonadism)^73^ and *NKX6-2*,^74^ although to date these syndromes are only described with childhood onset.

### Very rare genes (*RNF216*, *TREM2*, *SNORD118*)

Gordon Holmes syndrome is a rare autosomal recessive disorder characterised by hypogonadotrophic hypogonadism and ataxia. It is usually accompanied by cognitive decline, diffuse leukoencephalopathy with cerebellar atrophy and chorea. Age of onset is variable and the disorder is caused by mutations in the gene *RNF216*. The combination of chorea and dementia is reminiscent of an autosomal recessive form of Huntington disease.^75^

Polycystic lipomembranous osteodysplasia with sclerosing leukoencephalopathy, also known as Nasu-Hakola disease, is a rare syndrome caused by autosomal recessive mutations in *TREM2* or *TYROBP*. The disorder usually presents in adulthood, with bone pain and pathological fractures due to bone cysts. Later, a severe neuropsychiatric syndrome develops due to a progressive leukoencephalopathy. It is most common in Japan and Finland.^76^ Different *TREM2* variants are also significant risk factors for Alzheimer disease.^77^

Labrune syndrome, also known as leukoencephalopathy with brain calcification and cysts, is a rare autosomal recessive disorder caused by mutations in *SNORD118*. Labrune syndrome most commonly presents in early life, but there are reports of patients presenting in their 50s. The syndrome consists of progressive dementia, with motor decline and seizures. Large areas of cystic degeneration and calcification occur with a symmetric leukoencephalopathy affecting the periventricular, deep and subcortical white matter.^78^ *SNORD118* is not well covered by next-generation sequencing, and in the right clinical context single-gene sequencing is recommended.

### The Queen Square Adult Leukodystrophy Group

The Queen Square Adult Leukodystrophy Group (QSALG) is a multidisciplinary special interest group with input from neuroinflammation, neurogenetics, inherited metabolic disease, cognitive neurology and neuroradiology. Since inception, the group has investigated 116 patients with a wide variety of white matter syndromes, referred from hospitals worldwide. In our experience, a diagnosis can be made in approximately half of patients (54). The most common diagnoses are found in online supplementary figure 1. The most common diagnosis reached is of small vessel disease, followed by *CSF1R* mutations, mitochondrial diseases, ALD and multiple sclerosis. The remainder of diagnoses were highly heterogeneous, with often only one or at most two cases for each disorder. There were 15 diagnoses that affected only one patient each, and these included CADASIL and CARASIL, *PLP1* mutations, Alexander disease and Fabry disease, VWM disease and familial British dementia. This review is representative of the diagnostic approach of the QSALG.

10.1136/jnnp-2018-319481.supp1 Supplementary data

### Diagnostic algorithm

**Figure 3:**
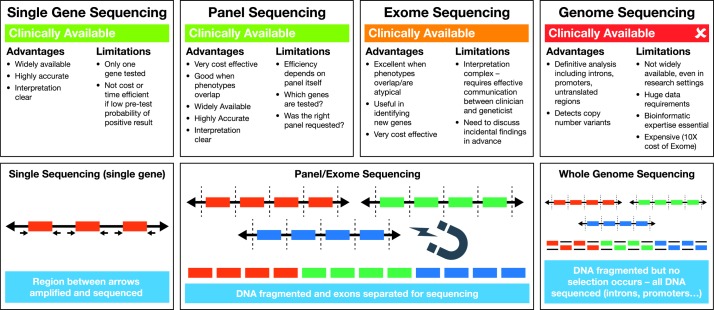
A schematic diagram to illustrate currently available sequencing technologies. Single-gene (Sanger) sequencing is widely available and is performed by amplifying coding regions of the gene of interest by PCR, before sequencing these regions. Introns and promoter regions are not routinely sequenced. Next-generation sequencing-based panel or exome sequencing is becoming more widely available clinically. In this technique, DNA is fragmented, and the exons of multiple genes of interest are selected out for sequencing. Although many genes are sequenced, again only the coding regions are selected. In whole genome sequencing, which is not routinely available to clinicians yet, the DNA is also fragmented, but all the resulting DNA is sequencing, without a selection step. This results in a large amount of data, as the coding regions, intergenic regions, introns and promoters of all human genes are sequenced. Advanced bioinformatics and computing technology are a required part of whole genome sequencing.

Figure 3 shows an algorithm that clinicians can use when evaluating adults presenting with a suspected leukoencephalopathy. As described above, initial assessment should focus on the exclusion of common acquired causes and severe small vessel disease (round 1). If these initial tests are negative and the patient is suspected to have a genetic disorder, then the first line of testing should include white cell enzyme activities, a VLCFA profile (in men), plasma cholestanol and bile alcohols and plasma amino acids, to exclude the classical leukodystrophies which can present in adulthood (round 2). This step is important, as enzyme replacement therapy and stem cell transplant are being explored as treatment options for some of these conditions.

**Figure 4:**
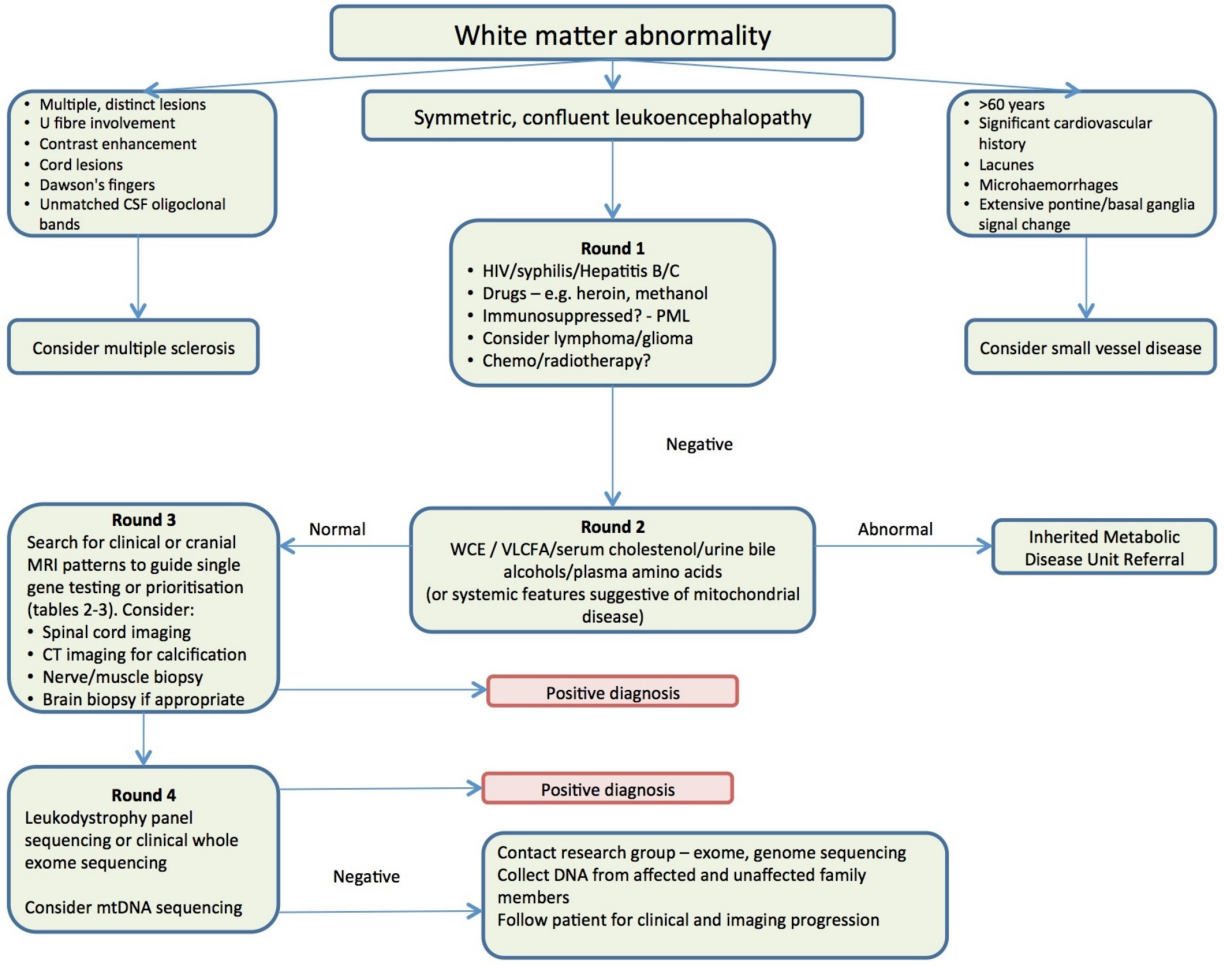
A recommended algorithm for the evaluation of adults with suspected inherited white matter disorders. CSF, cerebrospinal fluid; mtDNA, mitochondrial DNA; PML, progressive multifocal leukoencephalopathy; VLCFA, very long chain fatty acids; WCE, White cell enzymes.

If metabolic testing is not revealing, then the clinician should move on to genetic testing in the context of round 3 clinical or imaging patterns. A schematic illustrating the differences between sequencing approaches can be found in figure 4. Next-generation sequencing via *diagnostic panels* is becoming more widely available through regional genetic testing services, and these can be the most rapid and cost-effective route to diagnosis. It is worth determining which genes are covered by the panel requested, as each panel will differ depending on its design. If there is no access to a diagnostic panel, then consideration should be given to *single-gene* testing guided by the clinical and radiological phenotypes described above, and found in tables 1 and 2. It should be noted that single-gene testing is a low-yield route to diagnosis, and if a number of genes are sequenced the cost will quickly exceed that of a diagnostic panel. There are a small number of genes where a panel will not be sufficient for diagnosis, usually where copy number variants are a significant concern, and these would include *LMNB1* and *PLP1*. In these cases, clinical suspicion should guide directed testing.

If a panel-based approach is not successful but a genetic leukoencephalopathy is still considered likely, then consideration should be given to whether the disorder might be due to a mutation in the *mitochondrial genome* (not targeted by traditional panels). It may be necessary to contact a research group who can offer more extensive genetic testing including *whole exome or even whole genome sequencing*. In these cases, it is helpful to have obtained DNA from as many affected and unaffected family members as possible.

In our experience of difficult-to-diagnose patients who have already been extensively investigated, a focused exome panel led to a diagnosis in almost 30% of patients.^3^

## Discussion

Leukoencephalopathies are a heterogeneous group of disorders with a wide range of genetic and acquired causes. They are frequently difficult to diagnose, and patients with these disorders typically undergo a large number of expensive, time-consuming and often invasive tests over a long timeframe. This presents a significant burden to patients who usually are deteriorating from a severe and progressive neurological syndrome. The aim of the treating neurologist should be to make a definitive diagnosis, as this allows for better prognostication, more definitive family counselling and prevents further worry about the diagnosis. We hope that we have given a current useful, straightforward logical algorithm that others will find useful. It is clear that careful clinical and radiological assessment, in combination with early use of focused genetic testing, is needed to maximise the probability of making these diagnoses. However, even with this approach, more than half of patients currently do not receive a definitive diagnosis.^79^ This may be because the disorders are so heterogeneous or because there are still more genes to be described. Advances in genetic technology like whole genome sequencing may solve some cases, but only in combination with a thorough phenotypic approach.

Ultimately, these diagnoses are important because the development of novel therapies for these disorders depends primarily on forming cohorts of patients for clinical trials. HSCT from donors is a potential treatment for ALD, MLD and a number of other disorders. Recently, gene correction has also shown promise. In principle, gene correction (eg, through lentiviral transduction of the patient’s own cells) should be possible in most loss of function genetic disorders, but to show efficacy in rare diseases sufficient numbers of patients must be identified.^80^

As in 2014, the authors are happy to be contacted to discuss or see adult-onset patients with possible leukodystrophies or genetic leukoencephalopathies.
