# Supplementary figures and images for: Practical approach to the diagnosis of adult-onset leukodystrophies: an updated guide in the genomic era
Source: J Neurol Neurosurg Psychiatry. 2018 Nov 22;90(5):543–54. doi: 10.1136/jnnp-2018-319481 (PMC6581077; doi:10.1136/jnnp-2018-319481)

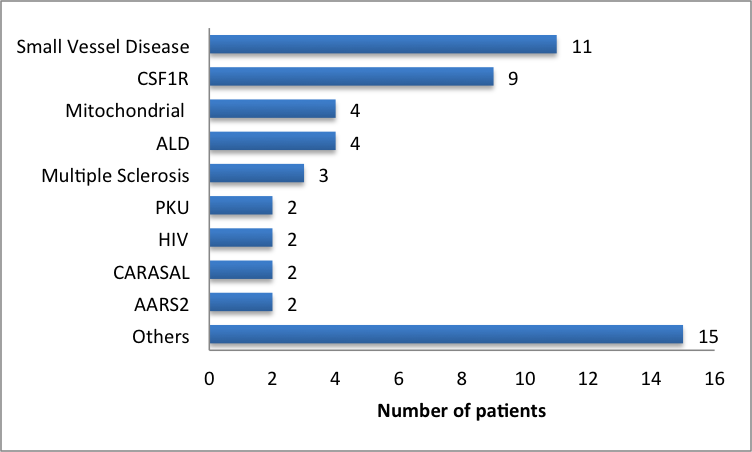

Supplement: Supplementary data [file jnnp-2018-319481supp001.png]
